# Endolymphatic Sac Enlargement in a Girl with a Novel Mutation for Distal Renal Tubular Acidosis and Severe Deafness

**DOI:** 10.1155/2012/605053

**Published:** 2012-08-27

**Authors:** Rink Nikki, Bitzan Martin, O'Gorman Gus, Nagel Mato, Torban Elena, Goodyer Paul

**Affiliations:** ^1^Pediatric Nephrology, The Montreal Children's Hospital, McGill University, Montreal, QC, Canada H3H 1P3; ^2^Pediatric Radiology, The Montreal Children's Hospital, McGill University, Montreal, QC, Canada H3H 1P3; ^3^Center for Nephrology and Metabolic Diseases, Albert-Schweitzer-Ring 32, 02943 Weisswasser, Germany; ^4^Department of Medicine, McGill University, Montreal, QC, Canada H3G 1Y6

## Abstract

Hereditary distal renal tubular acidosis (dRTA) is caused by mutations of genes encoding subunits of the H^+^-ATPase (ATP6V0A4 and ATP6V1B1) expressed in **α**-intercalated cells of the distal renal tubule and in the cochlea. We report on a 2-year-old girl with distal RTA and profound speech delay which was initially misdiagnosed as autism. Genetic analysis showed compound heterozygous mutations with one known and one novel mutation of the ATP6V1B1 gene; cerebral magnetic resonance imaging (MRI) revealed bilateral enlargement of the endolymphatic sacs of the inner ear. With improved cooperation, audiometric testing showed that hearing loss was most profound on the right, where endolymphatic sac enlargement was greatest, demonstrating a clear link between the degree of deafness and the degree of inner ear abnormality. This case indicates the value of MRI for diagnosis of inner ear involvement in very young children with distal RTA. Although citrate therapy quickly corrects the acidosis and restores growth, early diagnosis of deafness is crucial so that hearing aids can be used to assist acquisition of speech and to provide enough auditory nerve stimulation to assure the affected infants remain candidates for cochlear implantation.

## 1. Introduction

Distal renal tubular acidosis is characterized by a failure of mechanisms in the distal renal tubules that normally permits excretion of the daily hydrogen ion load; affected patients develop systemic acidosis, failure to thrive, and dissolution of bone mineral that leads to nephrocalcinosis or renal stones. Several hereditary forms of dRTA are caused by mutations in genes that encode the tubular transporter linked to distal hydrogen ion excretion. Specific missense mutations of the Cl^−^/HCO_3_ exchanger (AE1) gene (*SLC4A1*) expressed in alpha-intercalated cells cause isolated autosomal dominant type of dRTA in Europeans [1]. Specific *SLC4A1* mutations cause dRTA associated with hemolytic anemia or spherocytosis since the AE1/band 3 is also crucial for erythrocyte integrity [2]. Mutations in two different subunits of the alpha-intercalated cell luminal H^+^ATPase have been linked to recessive dRTA and sensorineural deafness [3]. H^+^ATPase is required both for renal tubular acidification and for H^+^ ion transport into the endolymph of the inner ear [4]. Children with homozygous mutations in the ATP6V1B1 gene have progressive hearing loss beginning in early childhood whereas homozygous mutations in the ATP6V0A4 gene were classically known to have milder or late-onset hearing loss, however there are also cases reported with severe early hearing loss associated with ATP6V0A4 mutations [5]. 

Inner ear morphological abnormalities such as the enlarged vestibular aqueduct syndrome also named large endolymphatic duct and sac syndrome (LEDS) can be found in a variety of different etiologies for hearing loss in children [6]. In dRTA with sensorineural hearing loss associations between an enlarged vestibular aqueduct (EVA) and both autosomal recessive genes (ATP6V0A4 and ATP6V1B1) have been made [7, 8], however a clear link between the degree of hearing loss and degree of EVA could not be established yet.

Recognition of primary dRTA is fairly straightforward for the pediatric nephrologist, since affected children have alkaline urine and absence of urinary ammonium ion, despite systemic acidosis. However, in infants, it may be difficult to determine whether there is associated sensorineural deafness and whether hearing loss is severe. This is a crucial issue, since it may prompt early hearing aid intervention and preserve enough auditory nerve function to make eventual cochlear implantation worthwhile. We report the case of a young girl with dRTA who is compound heterozygous for one known and one novel mutation in the ATP6V1B1 gene in whom inner ear involvement was initially uncertain but was firmly demonstrated by endolymphatic sac dilatation visible on magnetic resonance imaging. In this case the endolymphatic sac is clearly more enlarged on the side that has the more severe hearing loss. 

## 2. Case Report

A 17-month-old girl was admitted to the Montreal Children's Hospital because of failure to thrive over the previous five months. She was the first child of a nonconsanguineous couple, the father of Greek descent and the mother of French Canadian origin. The parents reported that she often appeared thirsty and woke up in the night to drink, soaking 6–8 diapers per day. She was not tolerating solid foods well but had no chronic diarrhea.

Her weight was 7.940 kg (*z*-score −3.2) and height was 77 cm (*z*-score −0.88). Her development was significantly delayed; she was barely able to sit and was unable to walk. She made only babbling sounds, was very irritable, and did not interact normally with others. 

Laboratory testing revealed a normal anion gap metabolic acidosis (bicarbonate = 14.7 mmol/L) with hypokalemia (K^+^ = 2.6 mmol/L). Other serum values included: sodium = 133 mmol/L, chloride = 108 mmol/L, calcium = 2.46 mmol/L, phosphate = 1.3 mmol/L, creatinine = 45 *μ*mol/L, and normal PTH (3.9 pmol/L). Red blood cell morphology was normal, but urine pH  (7.5) was inappropriately high. There was no evidence of proteinuria or glucosuria, and tubular reabsorption of phosphate (81%) was only marginally decreased. 

**Figure 1:**
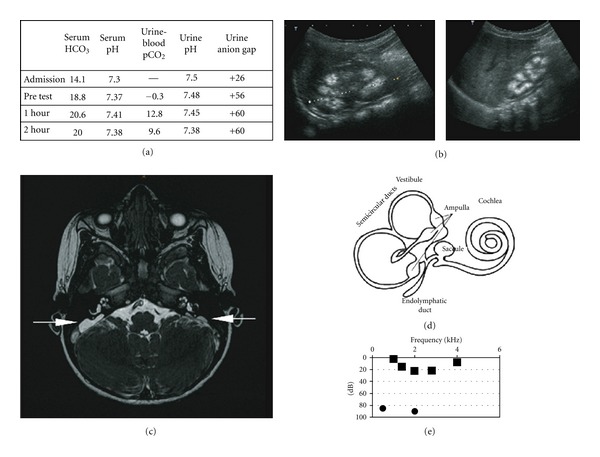
(a) Results of bicarbonate loading test with 1 mmol/kg NaHCO_3_ infusion. (b) Ultrasound of the left and right kidney with evidence of nephrocalcinosis. (c) Axial FIESTA cerebral MRI: bilateral enlargement of the endolymphatic sac (arrows), markedly larger on the right. (d) Schematic image of the inner ear. (e) Results of free field measurement and inserted right ear audiogram.

To formally assess distal renal tubular acidification, a sodium bicarbonate loading test was performed (1 mmol/kg of sodium bicarbonate was infused over 30 minutes). Distal RTA was confirmed by: (A) abnormally low urine minus plasma pCO_2_ < 30 mmHg; (B) positive urinary anion gap (UAG = U_Na^+^_ + U_K^+^_ − U_Cl^−^_) indicating absence of ammonium ion; (C) correction of serum bicarbonate from 18.8 to 20.6 meq/L with 1 mmol/kg NaHCO_3_ (Figure 1(a)). Her renal ultrasound showed bilateral nephrocalcinosis (Figure 1(b)). 

Although distal RTA was thought to explain her failure to thrive, her developmental delay was initially attributed to a familial form of autism since this diagnosis had been made in a maternal cousin and initial audiometry was inconclusive because of poor cooperation. However, cerebral magnetic resonance imaging revealed marked enlargement of the endolymphatic sac bilaterally. On the right, the sac measured 15 × 19 mm in transverse and craniocaudal dimensions, respectively (Figure 1(c)). Subsequent otoacoustic emission and audiograms (Figure 1(e)) confirmed absent hearing in the right ear (<80 dB) with residual hearing (2–25 dB) on the left. We sequenced all *ATP6V1B1* exons and identified a putative pathogenic mutation on each allele (IVS7 + G > T/p420fs434X). 

The patient started oral potassium citrate therapy (3 mmol citrate/kg per day). Her acidosis and hypokalemia resolved over the next 6 months, and she had good catch-up growth reaching weight *z*-scores of −1.12 (13% ile) by 2 years of age. Following interventions by the speech therapist, gross motor milestones, socialization, and speech have improved.

## 3. Discussion

Our patient presented with typical clinical signs of distal RTA, and further testing confirmed a defect in distal hydrogen ion secretion. However, her family history had led to a preliminary diagnosis of autism rather than deafness. Initial audiometry at 17 months of age was uninterpretable because of poor cooperation. Surprisingly, magnetic resonance imaging revealed dilatation of the endolymphatic sacs on both sides and clearly identified a primary pathology in the middle ear. Enlargement of the endolymphatic sacs has been reported in association with other forms of sensorineural hearing impairment, but in most (84%) there are coexistent cochlear and/or vestibular anomalies [9] (not present in our case). 

Genetic testing showed that the patient was a compound heterozygote for a novel mutation (c.1259insT) and a previously described mutation (c.687 + 1G > T) in the ATP6V1B1gene [10]. The c.1259 insertion produces a frameshift in exon 13 disrupting the entire C-terminal third of the transcript. The c.687 + 1 substitution lies within the intron following exon 7 and presumably disrupts splicing; this mutation has been reported in Greek patients from Cyprus [10]. 

Interestingly, our patient had severe dilatation of the right endolymphatic sac, where there was no apparent hearing below 80 decibels. Endolymphatic sac dilatation was significantly less on the left side where there was still some residual hearing. Thus, magnetic resonance imaging may be useful in dRTA both to identify the inner ear pathology associated with H^+^ATPase mutations and to gauge the local extent of inner ear damage. This is particularly important since cochlear dysfunction is progressive and is not reversed by the alkali therapy that corrects distal RTA. Affected children with residual hearing may benefit by early hearing aid intervention to optimize acquisition of speech and to assure ongoing stimulation of the auditory nerve. This may maximize the success of subsequent cochlear implantation at a later time.

## References

[1] Bruce LJ, Cope DL, Jones GK (1997). Familial distal renal tubular acidosis is associated with mutations in the red cell anion exchanger (band 3, AE1) gene. *Journal of Clinical Investigation*.

[2] Tanphaichitr VS, Sumboonnanonda A, Ideguchi H (1998). Novel AE1 mutations in recessive distal renal tubular acidosis. Loss-of-function is rescued by glycophorin A. *Journal of Clinical Investigation*.

[3] Stover EH, Akil I, Al-Sabban EA (2002). Novel ATP6V1B1 and ATP6V0a4 mutations in autosomal recessive distal renal tubular acidosis with new evidence for hearing loss. *Journal of Medical Genetics*.

[4] Karet FE, Finberg KE, Nelson RD (1999). Mutations in the gene encoding B1 subunit of H^+^-ATPase cause renal tubular acidosis with sensorineural deafness. *Nature Genetics*.

[5] Vargas-Poussou R, Mouillier P, Le Pettier N (2006). Genetic investigation of autosomal recessive distal renal tubular acidosis: evidence for early sensorineural hearing loss associated with mutations in the ATP6V0A4 gene. *Journal of the American Society of Nephrology*.

[6] Berrettini S, Neri E, Forli F (2001). Large vestibular aqueduct in distal renal tubular acidosis: high-resolution MR in three cases. *Acta Radiologica*.

[7] Andreucci E, Bianchi B, Carboni I (2009). Inner ear abnormalities in four patients with dRTA and SNHL: clinical and genetic heterogeneity. *Pediatric Nephrology*.

[8] Shinjo Y, Kaga K, Igarashi T (2005). Distal renal tubular acidosis associated with large vestibular aqueduct and sensorineural hearing loss. *Acta Oto-Laryngologica*.

[9] Davidson HC, Harnsberger HR, Lemmerling MM (1999). MR evaluation of vestibulocochlear anomalies associated with large endolymphatic duct and sac. *American Journal of Neuroradiology*.

[10] Feldman M, Prikis M, Athanasiou Y, Elia A, Pierides A, Deltas CC (2006). Molecular investigation and long-term clinical progress in Greek Cypriot families with recessive distal renal tubular acidosis and sensorineural deafness due to mutations in the ATP6V1B1 gene. *Clinical Genetics*.

